# Propofol Ameliorates ox-LDL-Induced Endothelial Damage Through Enhancing Autophagy via PI3K/Akt/m-TOR Pathway: A Novel Therapeutic Strategy in Atherosclerosis

**DOI:** 10.3389/fmolb.2021.695336

**Published:** 2021-06-25

**Authors:** Hongyi Zhou, Fan Jiang, Yufang Leng

**Affiliations:** ^1^ The First School of Clinical Medicine, Lanzhou University, Lanzhou, China; ^2^ Department of Anesthesiology, Tongzhou Maternal and Child Health Hospital of Beijing, Beijing, China; ^3^ Department of General Medicine, Beijing Luhe Hospital, Capital Medical University, Beijin, China; ^4^ Department of Anesthesiology, The First Hospital of Lanzhou University, Lanzhou, China

**Keywords:** propofol, autophagy, atherosclerosis, PI3K/Akt/m-TOR, endothelial cells, oxidized low-density lipoprotein

## Abstract

**Objective:** Atherosclerosis (AS) represents a common age-associated disease, which may be accelerated by oxidized low-density lipoprotein (ox-LDL)-induced endothelial cell injury. This study aimed to investigate the effects of Propofol on ox-LDL-induced endothelial damage and the underlying molecular mechanisms.

**Methods:** Human umbilical vein endothelial cells (HUVECs) were exposed to ox-LDL to induce endothelial damage. HUVECs were pretreated with 0, 5, 25 and 100°μM Propofol, followed by exposure to 100°μg/ml ox-LDL for 24°h. Cell viability was assessed by cell counting kit-8 (CCK-8) assay. The expression of autophagy- and apoptosis-related proteins was detected via western blot. Autophagosome was investigated under a transmission electron microscope. After co-treatment with autophagy inhibitor Bafilomycin A1 or si-Beclin-1, cell apoptosis was detected by flow cytometry. Furthermore, under cotreatment with PI3K activator 740Y-P, PI3K/Akt/m-TOR pathway- and autophagy-related proteins were examined by western blot.

**Results:** With a concentration-dependent manner, Propofol promoted the viability of HUVECs exposed to ox-LDL, and increased LC3-II/I ratio and Beclin-1 expression, and decreased P62 expression. The formation of autophagosome was enhanced by Propofol. Furthermore, Propofol treatment elevated Bcl-2/Bax ratio and lowered Caspase-3 expression. Bafilomycin A1 or si-Beclin-1 distinctly ameliorated the inhibitory effects of Propofol on apoptosis in ox-LDL-exposed HUVECs. Moreover, Propofol lowered the activation of PI3K/Akt/m-TOR pathway in HUVECs under exposure to ox-LDL. However, its inhibitory effects were weakened by 740Y-P.

**Conclusion:** Collectively, this study revealed that Propofol could ameliorate ox-LDL-induced endothelial damage through enhancing autophagy via PI3K/Akt/m-TOR pathway, which might offer a novel therapeutic strategy in AS.

## Introduction

Atherosclerosis (AS) is an age-associated chronic inflammatory disease induced by multiple factors (Kattoor et al., 2019). The exact cause of AS is still unclear, but the generally accepted “injury-response theory”. Endothelial damage is the initial link of AS. There are many factors that cause endothelial damage, among which oxidized low-density lipoprotein (ox-LDL) is the main factor (Kattoor et al., 2019). LDL, the main carrier of cholesterol in the blood, is a lipoprotein particle that carries cholesterol into peripheral tissue cells (Trpkovic et al., 2015). LDL is converted into ox-LDL after oxidative modification (Hartley et al., 2019). Evidence demonstrates that ox-LDL promotes vascular endothelial dysfunction and structural damage, which is involved in cardiovascular diseases, especially AS (Kattoor et al., 2019). Ox-LDL has been considered as a major marker of AS (Li et al., 2019). The endothelial damage model induced by ox-LDL has been widely utilized to explore the mechanism of AS. Ox-LDL affects the function of endothelial cells in many ways, thereby inducing the occurrence and development of AS (Chang et al., 2019). For instance, ox-LDL facilitates apoptosis and ameliorates viability for endothelial cells (Zhong et al., 2018). Thus, it is of importance to develop novel drugs like Propofol to ameliorate ox-LDL-induced endothelial damage.

Propofol (2,6-diisopropylphenol) is a commonly used intravenous anesthetics (Zitta et al., 2018). Propofol can relieve endothelial cell dysfunction through multiple mechanisms. For example, Propofol ameliorates P66shc expression in endothelial cells exposed to high glucose via Sirt1 (Wang et al., 2019a). It can improve endothelial damage via suppressing HMGB1 release (Feng et al., 2019). Furthermore, endothelial dysfunction and inflammatory response are relieved by Propofol through inhibiting PP2A expression (Wu et al., 2017). However, it remains unclear whether Propofol can improve endothelial damage caused by ox-LDL. Previously, Propofol can increase the movement and angiogenesis of endothelial cells via enhancing autophagy (Chang et al., 2015). However, the specific molecular mechanism still needs further exploration. Autophagy plays a critical physiological role in eukaryotic cells (Dikic, 2017). In the case of energy or nutrient deficiency, it can prevent cell damage, thereby adapting to the ever-changing environment (Schaaf et al., 2019). PI3K is a phosphatidylinositol kinase widely distributed in the cytoplasm (Zhan et al., 2020). It is an important signal transduction molecule in the cells (Jing et al., 2019). It can be activated by a variety of extracellular signals such as hormones, growth factors and cytokines, thereby converting phosphatidylinositol 4,5-bisphosphate into phosphatidylinositol 3,4,5-triphosphate, which binds to the PH domain in the N-terminal of Akt (Zegeye et al., 2018). Akt is finally activated by transferring from near the cell membrane to the cytoplasm. m-TOR, as a key downstream signal of PI3K/Akt, is activated after being phosphorylated (Wang et al., 2018b). mTOR negatively regulates autophagy. In the process of autophagosome formation and maturation, there are many autophagy-related markers involved, like light chain 3 (LC3) composed of LC3-I and LC3-II two forms, Beclin-1 and P62 (Wang et al., 2019a). It has been found that moderate autophagy of endothelial cells inhibits the development of AS. However, excessive autophagy may induce AS progression (Wang et al., 2018a). In this study, we used ox-LDL to establish a vascular endothelial cell injury model. The effects of Propofol on endothelial damage were investigated. Moreover, their underlying molecular mechanisms were explored in depth.

## Materials and Methods

### Cell Culture and Treatment

HUVECs (American Type Culture Collection, United States) were cultured in the DMEM medium (GIBCO, United States) plus 10% fetal bovine serum (044; GIBCO) in a 5% saturated humidity incubator at 37°C. HUVECs were separately stimulated by different concentrations (0, 10, 20, 50, 100 and 150°μg/ml) of ox-LDL (Cat. No.YB-002; Yiyuan biotechnology, China) for 24°h. Furthermore, HUVECs were exposed to 100°μg/ml ox-LDL for 6°h, 12°h and 24°h, respectively. HUVECs were pretreated with different concentrations of Propofol (0, 5, 25 and 100°μM), 100°μM Bafilomycin A1 (Gene operation, United States) or PI3K activator 740Y-P, followed by exposure to 100°μg/ml ox-LDL for 24°h.

### Flow Cytometry Assay

Apoptosis was detected following the instructions of Annexin V / PI Flow Double Staining Detection Kit (Nanjing KGI Biotechnology Development Co., Ltd., China). The cell concentration was adjusted to 1 × 10^5^°cells/well, and 2°ml / well was inoculated in a 6-well culture plate, which was cultured in a 5°C and 5% CO_2_ incubator. After treatment with different conditions, the cells of each group were collected in a 6-well plate (1 × 10^6^°cells/ml). 1°ml of the cell suspension was centrifuged at 1,000°rpm at 4°C for 10°min. Following discarding the supernatant, 1°ml of pre-cooled PBS was added, and gently shook to suspend the cells. After washing the suspended cells twice, the cells were resuspended in 200°μl 1 × Binding Buffer. 5°μl Annexin V-FITC was added and incubated for 15°min in the dark. Then, cells were incubated with 10°μl PI under dark conditions. Finally, early and late apoptosis was monitored using flow cytometry.

### Cell Viability Assay

HUVECs were seeded in 96-well plates at 1 × 10^3^/well and pre-cultured for 12°h. After 24°h of incubation under different conditions, 10°μl Cell Counting Kit-8 (CCK-8) solution (Dojindo, Japan) was added to each well and incubated in a 37°C incubator for 2°h. The absorbance of each well was weighed up at 450°nm with a microplate reader.

### Western Blot

Treated HUVECs or tissues were lysed by RIPA lysates (P0013B, Beyotime, Beijing). After being processed by the ultrasonic cell disruptor, the sample was centrifuged at 12,000°g at 4°C for 5°min on the ice. The supernatant was collected in a new EP tube. The BCA protein assay kit (#23227; Thermo, United States) was utilized to evaluate the protein concentration. The protein sample was separated by SDS-PAGE gel electrophoresis and transferred to the PVDF membrane. The membrane was sealed with 5% skimmed milk powder (232100-500°g, BD, United States) at room temperature for 2°h. Following being incubated with the primary antibodies against PI3K (1:1,000; ab32089; Abcam, United States), p-PI3K (1:1,000; ab86714; ab32089), Akt (4298; CST, United States), p-Akt (12694), mTOR (2972; Cell Signaling, United States), p-mTOR (2971; Cell Signaling), LC3-I / II (1:1,000; ab62721; Abcam), Beclin-1 (1:1,000; ab210498; Abcam), P62 (1:1,000; ab211324; Abcam), Bcl-2 (1:1,000; ab218123; Abcam), Bax (1:1,000; ab3191; Abcam), Caspase-3 (1:1,000; ab179517; Abcam) and β-actin (1:1,000; ab115777; Abcam) overnight at 4°C, samples were incubated with horseradish Peroxidase (HRP) labeled goat anti-rabbit (ZB-2301; ZSGB-BIO, Beijing, China) or anti-mouse (ZB-2305; ZSGB-BIO) IgG at room temperature for 2°h. The expression of target proteins was quantified by ImageJ software.

### Lactate Dehydrogenase Release Assay

Cells were lysed by RIPA lysates (Beyotime). LDH release levels were monitored using a LDH cytotoxicity assay kit (Beyotime) in line with the specification. The results were analyzed by the Automatic analyzer (AU600; Olympus, Japan).

### Transmission Electron Microscope

After centrifugation, HUVECs were fixed with 5% glutaraldehyde. Then, cells were fixated with 1% osmium acid-1.5% potassium ferrocyanide for 2°h, and rinsed with PBS. Alcohol-acetone gradient was used for dehydration. The samples were embedded by epoxy resin 618 embedding agent. After the ultrathin sections were made, the sections were stained with uranyl acetate and lead citrate. The morphology of autophagosomes were observed under transmission electron microscope (Hilips, Netherlands).

### Transfection

HUVECs (3.0 × 10^5^) were seeded onto a 6-well plate. After 24°h, 2°μg si-Beclin-1 (5′-UUC​AAC​ACU​CUU​CAG​CUC​AUC​AUC​C-3′; GenePharma, China) and negative control (5′-UUC​UCC​GAA​CGU​GUC​ACG​UTT-3′; GenePharma, China) were transfected into HUVECs via Lipofectamine 2000 (Invitrogen, United States). After 6°h of transfection, a new medium was changed and continued to culture for 18°h. Then the cells were pretreated with Propofol for 2°h, and then treated with 100°μg/ml ox-LDL for 24°h. The transfection results were assessed by western blot.

### Atherosclerosis Mouse Models

Totally, twenty ApoE^-/-^ male mice with 8°weeks old were obtained from Shanghai Experimental Animal Center (China), which were housed in an environment with a day-night cycle of 12°h, a temperature of 22–25°C and a humidity of 50–70%. These animals were randomly separated into four groups: control, AS model, model + propofol and model + propofol + 740Y-P groups. To construct AS models, mice were fed by high fat diet for 12°weeks. Control mice were normally fed basic diet. At the 10th°week, AS mice were randomly separated into three groups: AS model, model + propofol and model + propofol + 740Y-P groups. In brief, mice in model + propofol group were injected with 75°mg/kg propofol intraperitoneally and those in model + propofol + 740Y-P group were injected with 75°mg/kg propofol and 22.4°mg/kg 740Y-P. Meanwhile, mice in AS model group were injected by normal saline. After 12°weeks, all mice were euthanized. This animal experiment gained approval by the Animal Ethics Committee of Lanzhou University (2020048).

### Hematein and Eosin

Fresh artery tissue specimens were fixed by 4% paraformaldehyde for 24°h. Then, the specimens were dehydrated and embedded. The tissues were cut into 4°μm thick. After being dewaxed, the sections were stained with hematoxylin and 0.5% eosin solution. Afterwards, the sections were mounted with neutral gum. The images were investigated under an optimal microscope (Olympus, Japan).

### Statistical Analysis

Statistical analyses were presented via GraphPad Prism 8.0. Each experiment was independently repeated three times. Measurement data are expressed as mean ± standard deviation. One-way analysis of variance (ANOVA) was utilized for comparisons between multiple groups. *p* < 0.05 was statistically significant.

## Results

### ox-LDL Stimulation Induces Apoptosis and Autophagy in HUVECs

**FIGURE 1:**
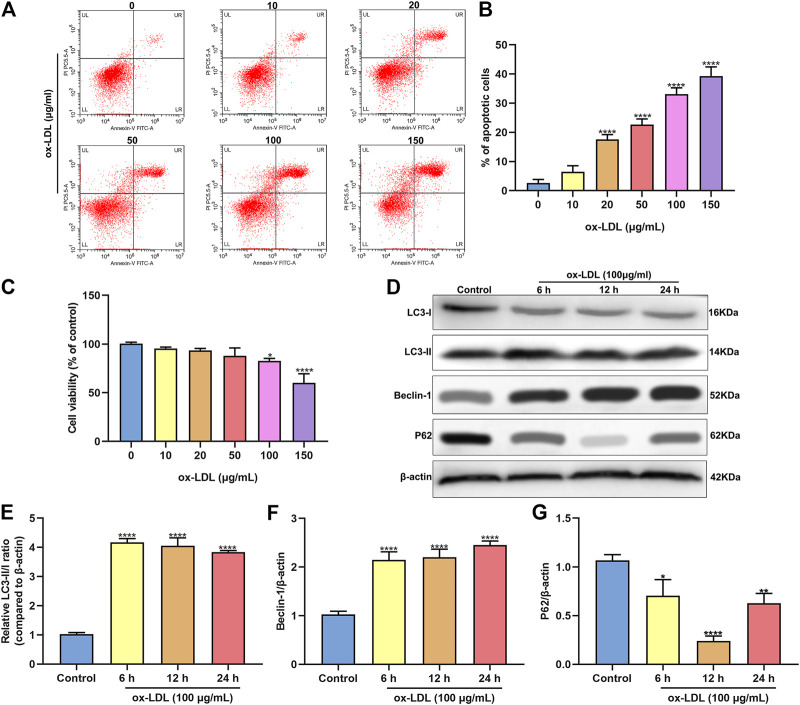
ox-LDL exposure heightens apoptosis and autophagy in HUVECs. **(A,B)** Flow cytometry assay results showing the apoptotic levels of HUVECs stimulated by 0, 10, 20, 50, 100 and 150°μg/ml ox-LDL for 24°h. **(C)** CCK-8 assay was utilized for assessment of the viability of HUVECs stimulated by 0, 10, 20, 50, 100 and 150°μg/ml ox-LDL for 24°h. **(D)** Representative images of western blots for LC3-I, LC3-II, Beclin-1 and P62 in HUVECs stimulated by 100°μg/ml ox-LDL for 6, 12 and 24°h. LC3- II / I ratio **(E)**, Beclin-1 **(F)** and P62 expression **(G)** levels were separately quantified. *p < 0.05; **p < 0.01 and ****p < 0.0001.

HUVECs were stimulated by 0, 10, 20, 50, 100 and 150°μg/ml ox-LDL for 24°h. When the concentration of ox-LDL reached 20°μg/ml, apoptotic levels were distinctly increased with a concentration-dependent manner (Figures 1A,B). As the concentration increased, the viability of HUVECs continued to decrease (Figure 1C). When the concentration of ox-LDL reached 100°μg/ml, cell viability was significantly decreased. Then, HUVECs were treated by 100°μg/ml ox-LDL. The expression of autophagy-related proteins was monitored via western blot. As a result, LC3 II/I ratio was markedly increased after stimulation with 100°μg/ml ox-LDL for 6, 12 and 24°h in comparison to control group (Figures 1D,E). In Figure 1F, Beclin-1 exhibited a significantly higher expression level in HUVECs treated with ox-LDL for 6, 12 and 24°h than controls. In comparison to controls, P62 protein levels were prominently lower in HUVECs induced by 100°μg/ml ox-LDL for 6, 12 and 24°h (Figure 1G).

### Propofol Enhances Autophagy in ox-LDL-Induced HUVECs

**FIGURE 2:**
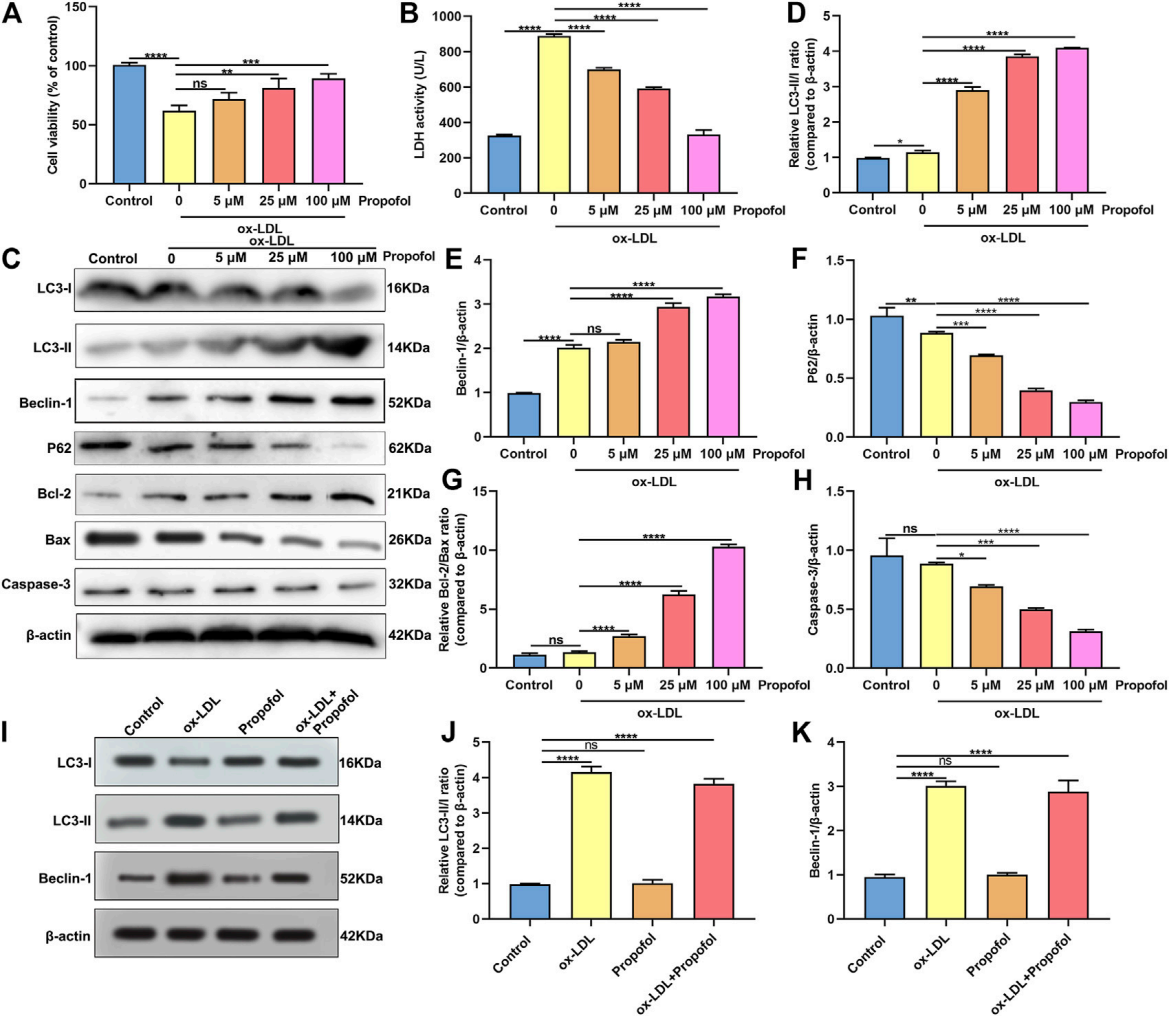
Propofol enhances autophagy in HUVECs under exposure to ox-LDL. **(A)** Cell viability was utilized for the evaluation of the viability of 100°μg/ml ox-LDL-induced HUVECs following treatment with 0, 5, 25 and 100°μM Propofol. **(B)** LDH release levels were determined in 100°μg/mL ox-LDL-exposed HUVECs following disposal with different concentrations of Propofol. **(C)** Representative of western blots for LC3-I, LC3-II, Beclin-1, P62, Bcl-2, Bax and Caspase-3. **(D)** LC3-II/I ratio, **(E)** Beclin-1, **(F)** P62, **(G)** Bcl-2/Bax and **(H)** Caspase-3 expression levels were quantified in 100°μM Propofol-treated HUVECs under exposure to 100°μg/ml ox-LDL. **(I-K)** Western blot for **(J)** LC3-II/I ratio and **(K)** Beclin-1 expression in HUVECs treated with 100°μg/ml ox-LDL and / or 100°μM Propofol. Ns: no statistical significance; *p < 0.05; **p < 0.01; ***p < 0.001 and ****p < 0.0001.

**FIGURE 3:**
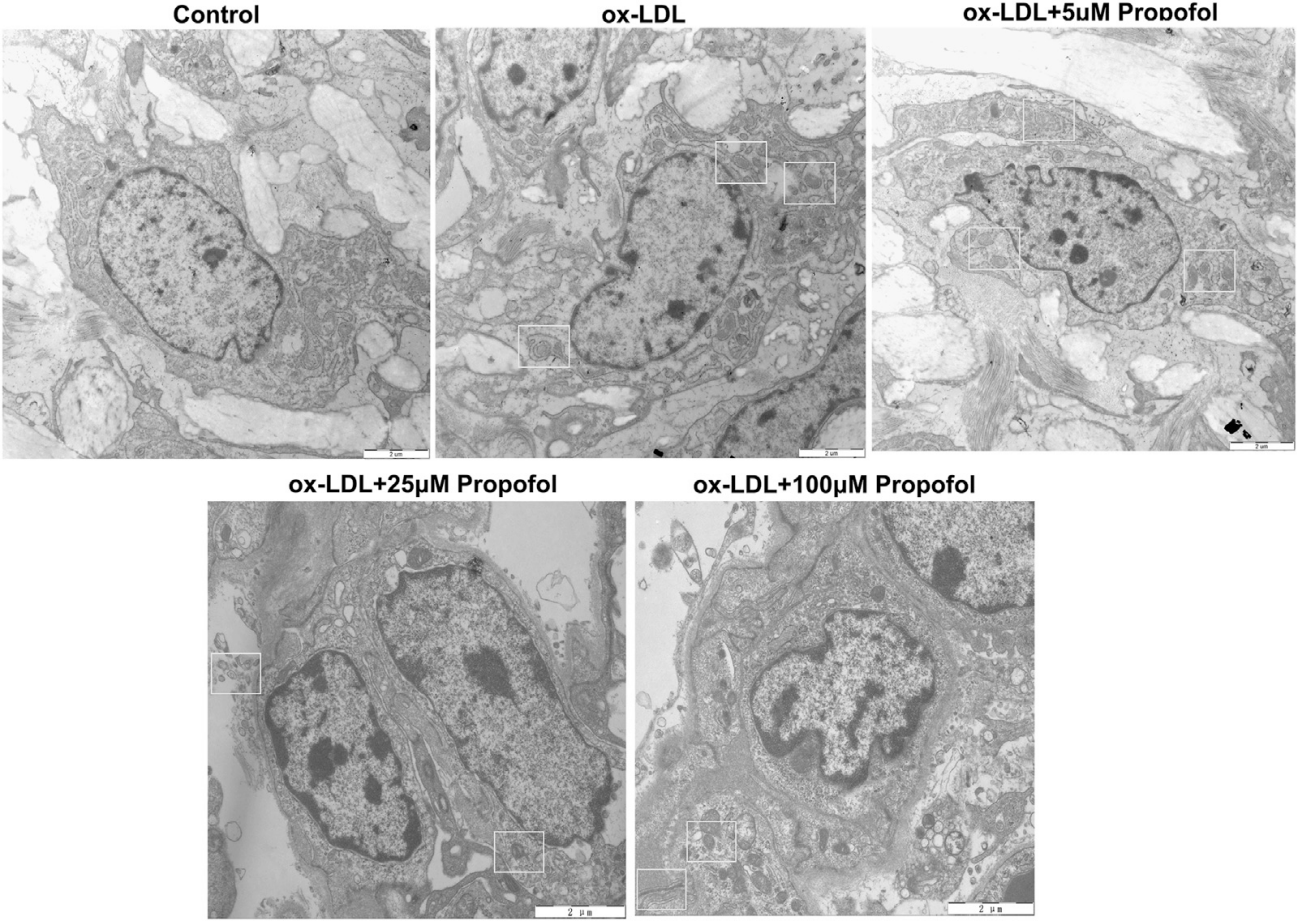
Transmission electron microscope for the autophagosomes in 100 μg/mL ox-LDL-exposed HUVECs treated with 0, 5, 25 and 100°μM Propofol (Bar = 2°μm). Autophagosomes are boxed.

Following treatment with different concentrations of propofol for 2°h, HUVECs were exposed to ox-LDL for 24°h. Our CCK-8 results suggested that 25 and 100°μM propofol markedly improved the viability of ox-LDL-stimulated HUVECs (Figure 2A). Moreover, we found that ox-LDL exposure distinctly induced LDH release in HUVECs (Figure 2B). However, propofol pretreatment distinctly ameliorated ox-LDL-induced LDH release in HUVECs. Additionally, we investigated whether propofol affected the autophagy induced by ox-LDL in HUVECs via western blot. Our data suggested that propofol distinctly increased the LC3 II/I ratio in HUVECs under exposure to ox-LDL, with a dose-dependent manner (Figures 2C,D). In Figure 2E. 25 and 100°μM propofol treatment prominently enhanced the expression of Beclin-1 in ox-LDL-induced HUVECs. As the concentration of propofol improved, P62 expression gradually decreased in HUVECs under exposure to ox-LDL (Figure 2F). The data suggested that Propofol can strengthen autophagy in ox-LDL-induced HUVECs. We also assessed the effects of Propofol on apoptotic levels in HUVECs under exposure to ox-LDL. Our data showed that Propofol distinctly augmented Bcl-2/Bax ratio in ox-LDL-exposed HUVECs, which was proportional to the concentrations of Propofol (Figure 2G). As shown in Figure 2H, Caspase-3 expression was gradually lowered by 0, 5, 25 and 100°μM Propofol in ox-LDL-induced HUVECs. These suggested that Propofol relieved apoptotic levels in HUVECs exposed to ox-LDL. The final concentration of Propofol was 100°μM for further experiments. We found that 100°μM Propofol did not alter the LC3 II/I ratio (Figures 2I,J) and Beclin-1 (Figure 2K) expression in non-ox-LDL-induced HUVECs, indicating that 100°μM Propofol did not affect the autophagy of normal HUVECs. Furthermore, the autophagosome formation was investigated under a transmission electron microscope. As shown in Figure 3, the autophagosome was discovered in ox-LDL-exposed HUVECs. Following treatment by Propofol, the amount of autophagosome was increased in HUVECs under exposure to ox-LDL.

### Propofol Ameliorates ox-LDL-Induced Damage of HUVECs via Enhancing Autophagy

**FIGURE 4:**
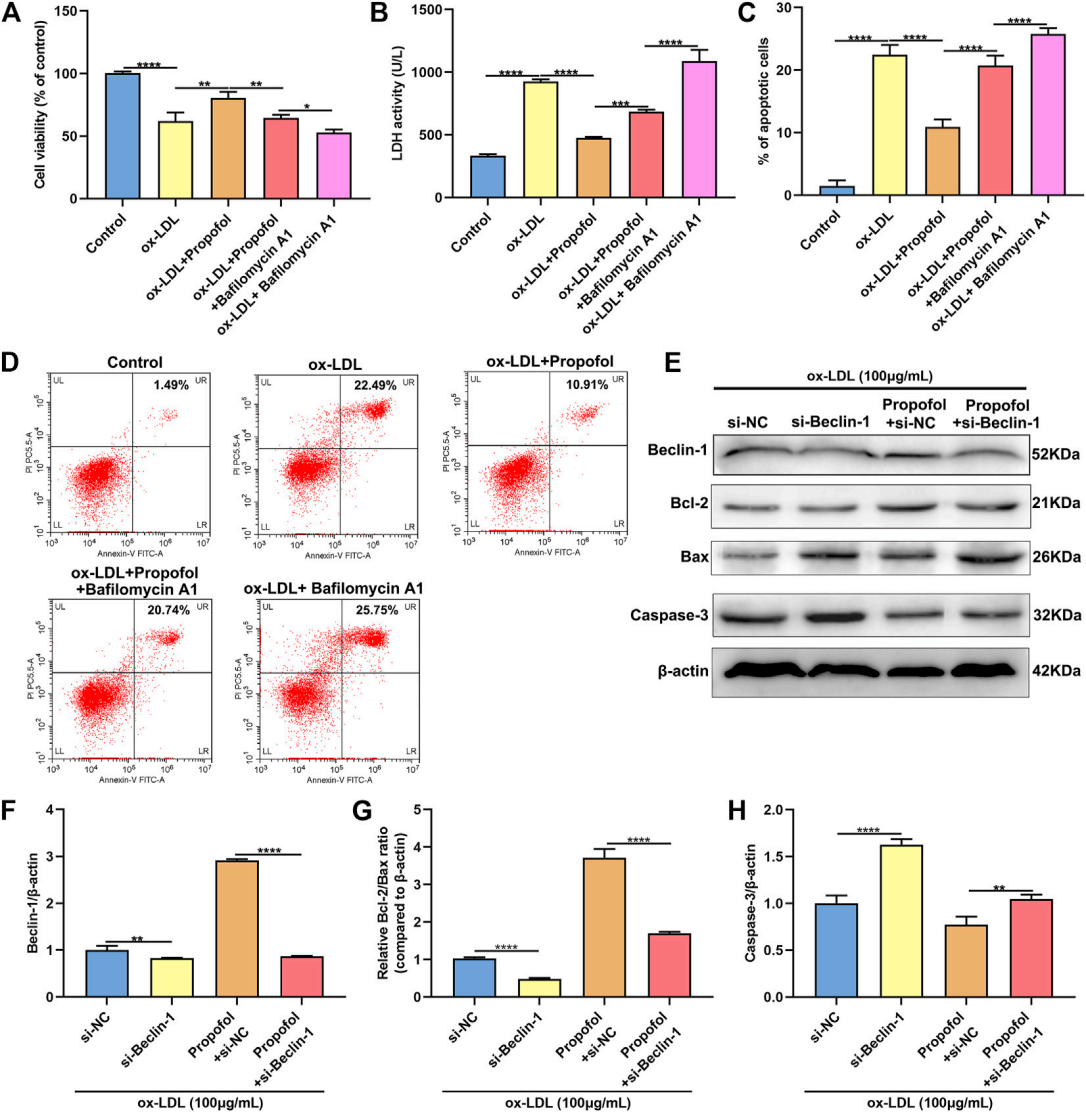
Propofol ameliorates ox-LDL-induced damage of HUVECs by enhancing autophagy. **(A)** The cell viability of 100°μg/ml ox-LDL-induced HUVECs treated with 100°μM Propofol and / or 100°μM Bafilomycin A1. **(B)** LDH release levels were examined in 100°μg/mL ox-LDL-induced HUVECs treated with 100°μM Propofol and / or 100°μM Bafilomycin A1. **(C, D)** Flow cytometry assay was presented to assess the apoptotic levels of 100°μg/mL ox-LDL-induced HUVECs treated with 100°μM Propofol and / or 100°μM Bafilomycin A1. **(E)** Representative images of western blot for Beclin-1, Bcl-2, Bax and Caspase-3 in 100°μg/ml ox-LDL-exposed HUVECs treated with si-Beclin-1 and / or 100°μM Propofol. **(F)** Beclin-1, **(G)** Bcl-2/Bax ratio and **(H)** Caspase-3 protein levels were quantified according to the western blot results. *p < 0.05; **p < 0.01; ***p < 0.001 and ****p < 0.0001.

**FIGURE 5:**
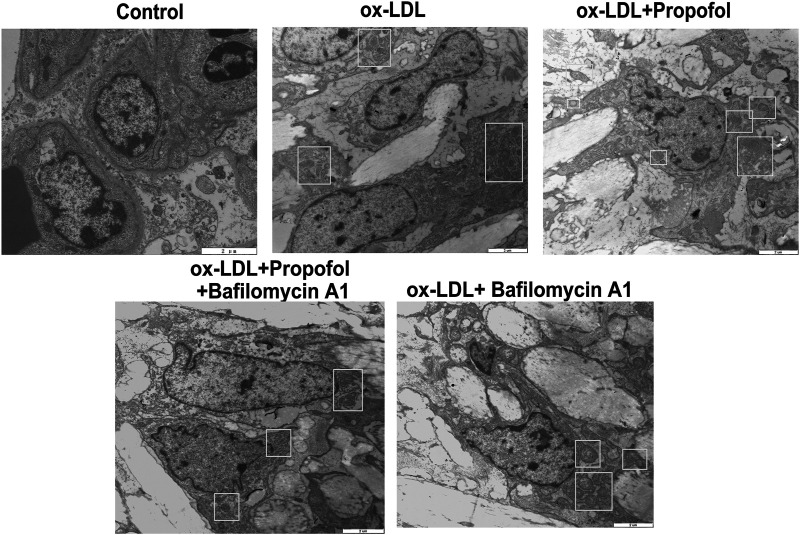
Transmission electron microscope for detection of the autophagosomes in 100°μg/ml ox-LDL-exposed HUVECs treated with 100°μM Propofol and / or Bafilomycin A1. Autophagosomes are boxed. Bar = 2°μm.

We further observed whether 100°μM Propofol might ameliorate ox-LDL-induced injury of HUVECs passing enhancing autophagy. The CCK-8 results suggested that the cellular viability of HUVECs was weakened by 100°μg/ml ox-LDL exposure. Nevertheless, its viability was ameliorated by 100°μM Propofol treatment. After co-treatment with 100°μM autophagy inhibitor Bafilomycin A1 and 100°μM Propofol, the viability of 100°μg/ml ox-LDL-induced HUVECs was significantly lowered than those treated with Propofol (Figure 4A). In Figure 4B. 100°μM Propofol treatment markedly weakened the LDH release of HUVECs under exposure to 100°μg/ml ox-LDL. However, this decrease was improved by 100°μM Bafilomycin A1. As shown in the flow cytometry assay results, the apoptotic levels were ameliorated by 100°μM Propofol for 100°μg/ml ox-LDL-induced HUVECs (Figures 4C,D). On the contrary, 100°μM Bafilomycin A1 co-treatment weakened the protective effects of 100°μM Propofol on 100°μg/ml ox-LDL-induced apoptosis in endothelial cells. This study further observed whether Beclin-1 knockdown affected the effects of Propofol on ox-LDL-induced damage in HUVECs. Si-Beclin-1 was transfected into ox-LDL-exposed HUVECs. The western blot confirmed that Beclin-1 was effectively suppressed (Figures 4E,F). Si-Beclin-1 transfection distinctly suppressed the enhancement in Beclin-1 expression treated by 100°μM Propofol treatment in 100°μg/ml ox-LDL-induced HUVECs. In Figure 4G, Bcl-2/Bax ratio was markedly inhibited via Beclin-1 knockdown in HUVECs under exposure to 100°μg/ml ox-LDL. Also, 100°μM Propofol significantly increased the Bcl-2/Bax ratio, which was distinctly lowered by si-Beclin-1 transfection in 100°μg/ml ox-LDL-induced HUVECs. Caspase-3 expression exhibited a significant increase following Beclin-1 knockdown in ox-LDL-exposed HUVECs (Figure 4H). This decrease in Caspase-3 expression induced by Propofol was reversed by silencing Beclin-1 in ox-LDL-induced HUVECs. In Figure 5, we detected the autophagosome expression in 100°μg/ml ox-LDL-exposed HUVECs. Our data confirmed that 100°μM Propofol increased the expression of autophagosome in 100°μg/ml ox-LDL-induced HUVECs. However, 100°μM Bafilomycin A1 cotreatment suppressed the effects of Propofol on the autophagosome expression. Collectively, Propofol could ameliorate ox-LDL-induced damage of HUVECs via enhancing autophagy.

### Propofol Boosts ox-LDL-Induced Autophagy via Inactivation of PI3K/Akt/m-TOR Pathway in HUVECs

**FIGURE 6:**
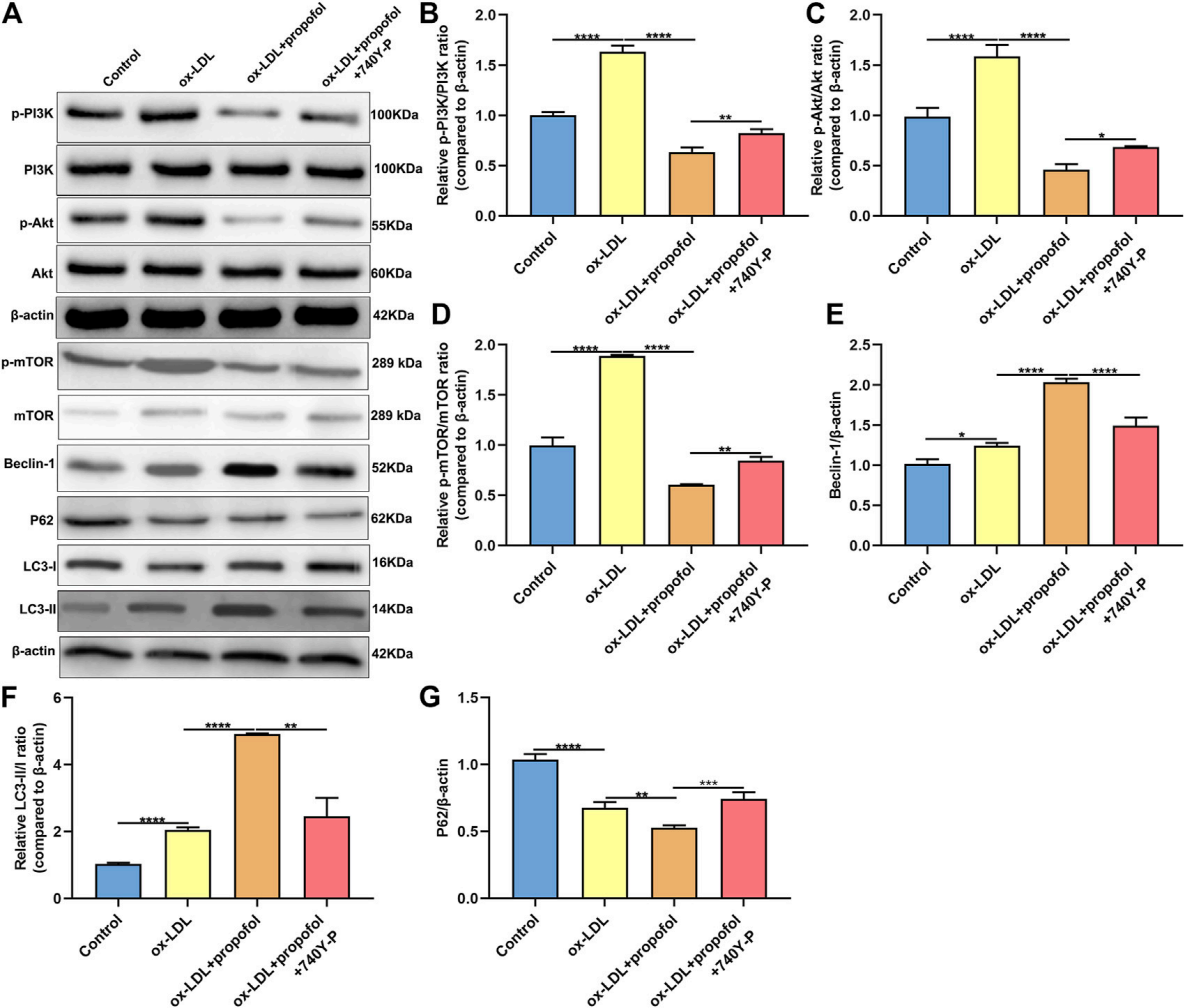
Propofol boosts autophagy via inhibiting PI3K/Akt/m-TOR pathway in ox-LDL-induced HUVECs. **(A)** Representative images of western blot for p-PI3K, PI3K, p-Akt, Akt, p-mTOR, mTOR, Beclin-1, P62, LC3-I and LC3-II in 100°μg/ml ox-LDL-exposed HUVECs treated with 100°μM Propofol and / or 100°μM 740Y-P. The quantification of **(B)** p-PI3K / PI3K ratio, **(C)** p-Akt / Akt ratio, **(D)** p-mTOR / mTOR ratio, **(E)** Beclin-1, **(F)** LC3-II / LC3-I ratio and **(G)** P62 expression based on the western blot results. *p < 0.05; **p < 0.01; ***p < 0.001 and ****p < 0.0001.

The molecular mechanisms of Propofol enhancing ox-LDL-induced autophagy were explored in depth. As shown in the western blot results, p-PI3K/PI3K ratio exhibited a distinctly higher level in HUVECs under exposure to ox-LDL than control group (Figures 6A,B). This increase was markedly ameliorated by Propofol treatment. However, the decrease in p-PI3K/PI3K ratio induced by Propofol was reversed by PI3K activator 740Y-P. In Figures 6C,D, p-Akt/Akt and p-mTOR/mTOR ratios were distinctly fortified in HUVECs exposed to ox-LDL compared to controls. Conversely, their ratios were prominently lowered by Propofol treatment. However, this decrease was significantly reversed by 740Y-P. Using the western blot, we evaluated the expression of autophagy-related proteins in ox-LDL-exposed HUVECs. These findings showed that Propofol distinctly enhanced Beclin-1 expression in ox-LDL-exposed HUVECs (Figure 6E). The enhancement was weakened by 740Y-P. The LC3 II/I ratio was distinctly elevated in ox-LDL-exposed HUVECs, which was reinforced by propofol treatment (Figure 6F). Nevertheless, this enhancement was reversed by 740Y-P treatment. The P62 expression was also examined. As shown in Figure 6G, propofol markedly lowered P62 expression in ox-LDL-exposed HUVECs, which was reversed by 740Y-P. Taken together, Propofol could boost autophagy through suppressing PI3K/Akt/m-TOR pathway in ox-LDL-induced HUVECs.

### Propofol Ameliorates Endothelial Damage and Promotes Autophagy in Atherosclerosis Mouse Models

**FIGURE 7:**
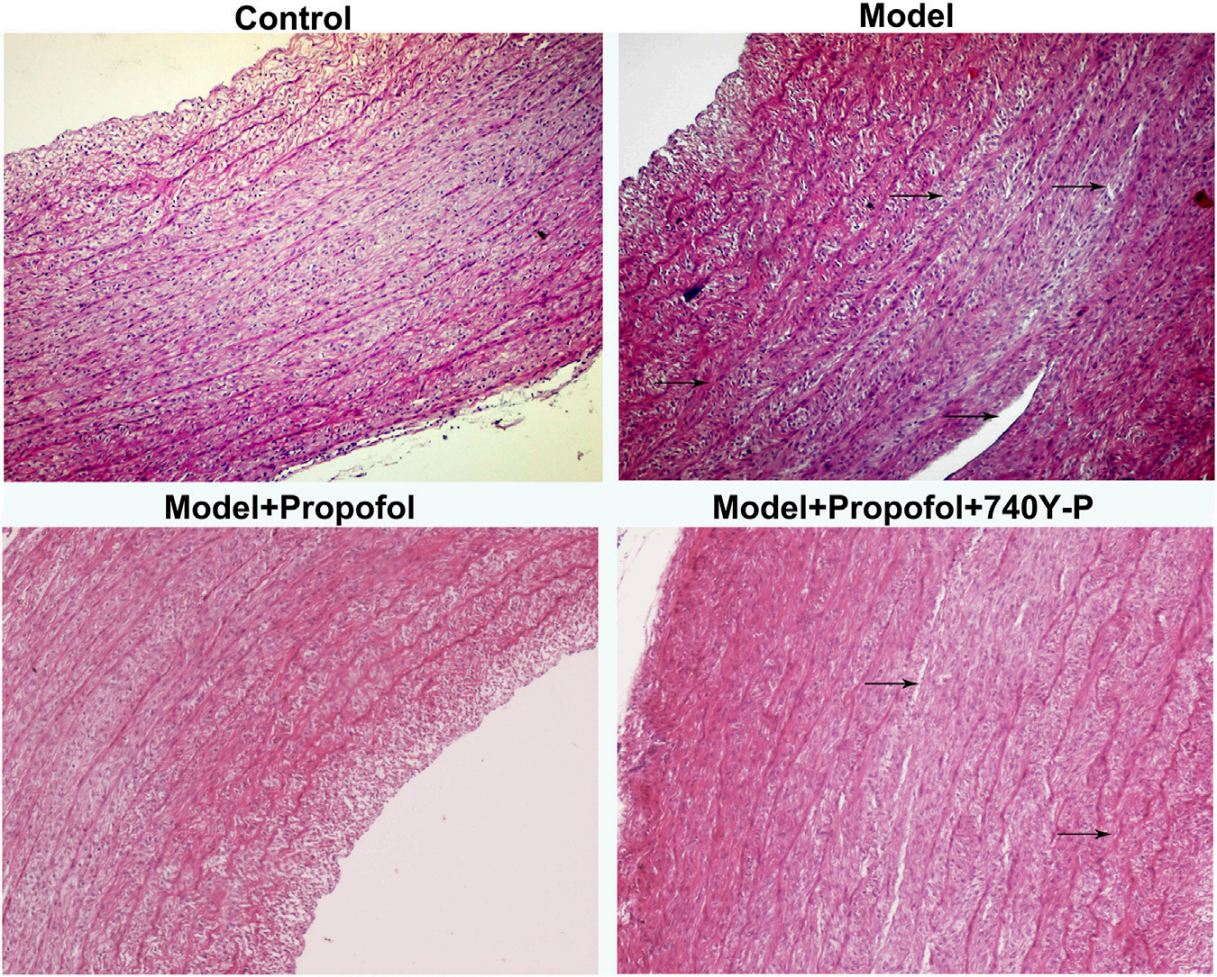
H & E staining for the endothelial damage in AS mouse models treated with Propofol and / or 740Y-P. The arrow points to the lesion. Bar = 20°μm.

**FIGURE 8:**
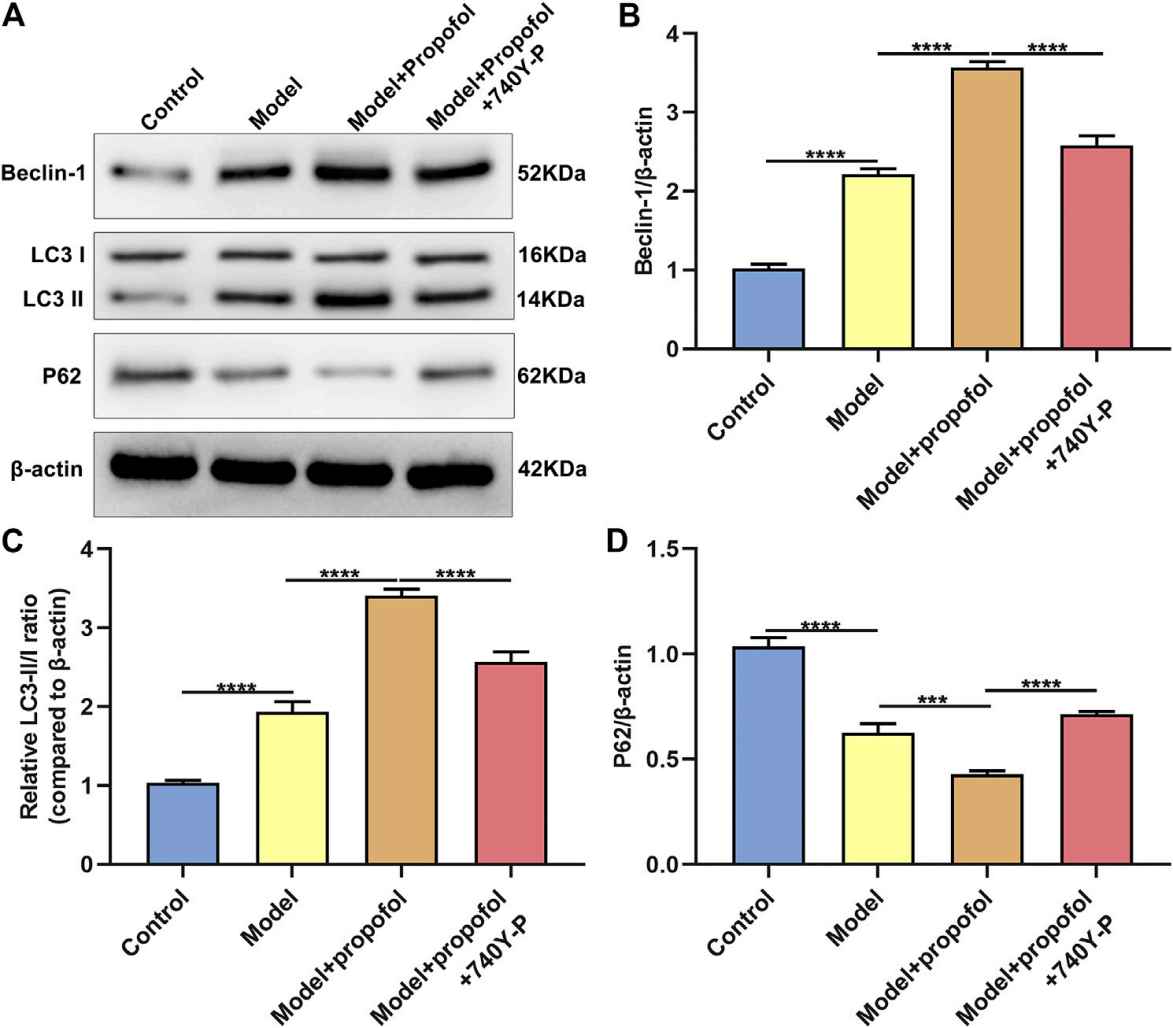
Propofol boosts autophagy in AS mouse models. **(A)** Representative images of western blot for Beclin-1, LC3II/I and P62 proteins in arterial tissues of control model mice, model mice treated with Propofol and model mice treated with Propofol and 740Y-P. The quantification of **(B)** Beclin-1, **(C)** LC3II/I and **(D)** P62 proteins based on the western blot results. ***p < 0.001 and ****p < 0.0001.

AS mouse models were constructed in this study. H & E staining results showed that mice in the model group had severe arterial endothelial injury compared to controls (Figure 7). Propofol treatment significantly ameliorated endothelial injury in AS mouse models. However, 740Y-P cotreatment partially weakened the therapeutic effects of propofol. Autophagy-related proteins including Beclin-1, LC3II/I and P62 were detected in arterial tissues by western blot (Figure 8A). Our data showed that Propofol treatment significantly increased the expression of Beclin-1 (Figure 8B), LC3II/I (Figure 8C) as well as decreased P2 expression (Figure 8D) in arterial tissues of AS mouse models. However, 740Y-P cotreatment partially weakened the effects of propofol on autophagy in arterial tissues of AS mouse models. Collectively, Propofol might ameliorate endothelial damage and promote autophagy in AS mouse models.

## Discussion

Endothelial cells are the first barrier of the vascular system against body damage, and the destruction of endothelial homeostasis is one of the early stages of AS (Eelen et al., 2015; Gimbrone and García-Cardeña, 2016; Paone et al., 2019). Endothelial cells are arranged in a single layer in the inner layer of blood vessels. Endothelial cell damage is recognized as one of the pathological characteristics of AS. As a highly selective permeable barrier between circulating blood and tissues, intact endothelium inhibits the adhesion of monocytes and platelets and maintains blood vessel homeostasis. ox-LDL is one of the risk factors of AS (Messner and Bernhard, 2014; Esse et al., 2019; Maguire et al., 2019). At present, vascular endothelial cells under exposure to ox-LDL are commonly used as cellular models for studying AS pathogenesis. Due to the increasing incidence of AS, there is an increasingly urgent need to study the molecular mechanisms at the cellular levels. In this study, we conducted an ox-LDL-induced endothelial injury cellular model. These results showed that Propofol ameliorated the endothelial damage induced by ox-LDL via enhancing autophagy and suppressing the PI3K/Akt/m-TOR pathway activation.

Apoptosis of vascular endothelial cells is one of the key signs of endothelial injury, which is closely related to the degree of endothelial damage and the severity of AS (Paone et al., 2019). Endothelial cell apoptosis can lead to local lipid deposition, thereby eventually developing AS. Prevention of endothelial cell apoptosis as a new treatment for AS has received greater attention. Consistent with previous research, our data showed that exposure to ox-LDL may stimulate the apoptosis of HUVECs. Furthermore, Propofol ameliorated inflammatory response of human cerebral microvascular endothelial cells (Ding et al., 2019). But Propofol significantly relieved the apoptosis-induced by ox-LDL exposure. Previously, Propofol can induce apoptosis of endothelial cells via various factors such as angiotensin II (Zhang et al., 2018), lipopolysaccharide (Lv et al., 2017), high glucose (Wang et al., 2019a), nitrosative stress (Chen et al., 2013) and hydrogen peroxide (Wang et al., 2007). Bcl-2 and Caspase gene families play an important role in the process of cell apoptosis. Herein, Propofol distinctly increased the Bcl-2/Bax ratio and lowered Caspase-3 expression in ox-LDL-exposed HUVECs. Hence, Propofol exhibited an inhibitory effect on the apoptosis of ox-LDL-exposed HUVECs.

Autophagy, an evolutionary conservative self-degradation process, is an important defense mechanism of cells under internal and external stimulation (He et al., 2020). It forms characteristic autophagosomes to encapsulate excess or damaged intracellular components and transport them to lysosomes degrade, thereby maintaining the stability of the cell’s environment. Previous studies have shown that ox-LDL can induce autophagy in HUVECs (Wang et al., 2019b). The activation of autophagy promotes the degradation of ox-LDL, which is a self-protection process of cells (Zhang et al., 2017). Our research also found that HUVECs treated with 100°μg/ml ox-LDL can increase LC3-II/I ratio and Becin-1 expression, and decrease P62 expression. Increased levels of autophagy are beneficial to enhance the tolerance of vascular endothelial cells to stress and survival (Schaaf et al., 2019). Transmission electron microscope results confirmed the increased number of autophagosomes after treatment with Propofol in HUVECs exposed to ox-LDL. Furthermore, Propofol augmented Beclin-1 expression and LC3 II/I ratio as well as lowered P62 expression in ox-LDL-exposed HUVECs. These data manifested that Propofol can boost ox-LDL-induced autophagy in HUVECs. Consistent with previous studies, Propofol could enhance autophagy of HUVECs (Chang et al., 2015). To further analyze whether Propofol ameliorated ox-LDL-induced endothelial damage via enhancing autophagy in HUVECs, HUVECs were treated or transfected with autophagy inhibitor Bafilomycin A1 or si-Beclin-1. As a result, Bafilomycin A1 or si-Beclin-1 distinctly lowered the inhibitory effects of Propofol on apoptosis induced by ox-LDL, suggesting that Propofol could suppress apoptosis of ox-LDL-exposed HUVECs via heightening autophagy.

The kinase mTOR is the main negative regulator of autophagy. It receives signals from different signal transduction pathways, which is the downstream target of the PI3K/AKT pathway. Research has shown that multiple signal pathways could participate in the regulation of autophagy in vascular endothelial cells. Among them, the PI3K/Akt/mTOR pathway is critical for autophagosome formation (Zheng et al., 2019). We tested the expression changes of related indicators in the PI3K/Akt/mTOR pathway in HUVECs treated with ox-LDL. These data suggested that ox-LDL promoted the activation of PI3K/Akt/mTOR pathway in HUVECs. However, Propofol decreased the activation of PI3K/Akt/mTOR pathway in ox-LDL-induced HUVECs, thereby enhancing the autophagy. As previous studies, Propofol could suppress renal (Wei et al., 2019) and hepatic (Wei et al., 2017) ischemia-reperfusion damage via this pathway. Our findings revealed that Propofol induced autophagy of HUVECs under exposure to ox-LDL partly via the inactivation of PI3K/Akt/mTOR pathway.

## Conclusion

Collectively, this study manifested that Propofol could ameliorate ox-LDL-induced endothelial damage via suppressing apoptosis and enhancing autophagy. Furthermore, Propofol inactivated the PI3K/Akt/m-TOR pathway in HUVECs under exposure to ox-LDL, thereby heightening the autophagy levels. Thus, Propofol could become a promising novel drug to relieve ox-LDL-induced endothelial damage.

## Data Availability

The original contributions presented in the study are included in the article/Supplementary Material, further inquiries can be directed to the corresponding author.

## Abbreviations

HUVECs: human umbilical vein endothelial cells; ox-LDL: oxidized low-density lipoprotein; LDL: low-density lipoprotein; AS: atherosclerosis; LDH: lactate dehydrogenase; ANOVA: one-way analysis of variance
